# Hepatitis A Hospitalization Costs, United States, 2017

**DOI:** 10.3201/eid2605.191224

**Published:** 2020-05

**Authors:** Megan G. Hofmeister, Shaoman Yin, Maria V. Aslam, Eyasu H. Teshale, Philip R. Spradling

**Affiliations:** Centers for Disease Control and Prevention, Atlanta, Georgia, USA

**Keywords:** hepatitis A, disease outbreaks, hospital costs, Healthcare Cost and Utilization Project, United States, viruses, vaccine-preventable diseases, hepatitis A virus

## Abstract

The United States is in the midst of unprecedented person-to-person hepatitis A outbreaks. By using Healthcare Cost and Utilization Project data, we estimated the average costs per hepatitis A–related hospitalization in 2017. These estimates can guide investment in outbreak prevention efforts to stop the spread of this vaccine-preventable disease.

The introduction of hepatitis A vaccine has dramatically changed the epidemiology of hepatitis A in the United States. After vaccine licensure in 1995, hepatitis A incidence declined substantially; 3,366 hepatitis A cases were reported nationally in 2017 (*1*).

During July 1, 2016–February 7, 2020, state health departments publicly reported >31,000 outbreak-associated cases, primarily affecting persons who use drugs and persons experiencing homelessness, in the largest person-to-person hepatitis A outbreaks in the postvaccine era (*2*). More than 18,900 (61%) outbreak-associated patients have reportedly been hospitalized in these outbreaks (*2*). As these unprecedented outbreaks continue, we sought to estimate the average direct medical costs per hepatitis A–related hospitalization, which can be used to guide investment in outbreak prevention efforts.

We analyzed data from the 2017 Healthcare Cost and Utilization Project National Inpatient Sample (NIS). NIS, a 20% stratified sample of discharges from US community hospitals, is the largest publicly available all-payer inpatient database in the country (*3*). We considered a hospitalization to be hepatitis A–related if it included codes B15.0 or B15.9 from the International Classification of Diseases, 10th Revision, Clinical Modification, as any of the 30 listed diagnosis codes. We converted the total hospital charges into cost estimates (in 2017 US dollars) by multiplying total charges with 2017 hospital-specific cost-to-charge ratios (*4*), then estimated the average cost of hospitalization, SD, and 95% CI on the basis of the NIS survey sampling design. We multiplied the average costs by the number of patients hospitalized for outbreak–associated hepatitis A to generate an estimate of the preventable economic burden of hospitalizations in the ongoing person-to-person outbreaks (*2*).

We examined hepatitis A–related hospitalizations in the 2017 NIS dataset for evidence of associated liver transplantation (procedure codes 0FY00Z0, 0FY00Z1, and 0FY00Z2 from the International Classification of Diseases, 10th Revision, Clinical Modification, listed as any of the 15 procedure codes). Because the unweighted number of hospitalizations associated with liver transplantation was <10, we included such hospitalizations in the analysis but did not report them as a separate category (*5*).

Overall, the average costs per hepatitis A–related hospitalization in the United States in 2017 were $16,232 (SD $602; 95% CI $15,052–$17,411). The average costs ranged from $12,921 (SD $1,443; 95% CI $10,091–$15,750) in the West North Central Census Division to $19,680 (SD $1,932; 95% CI $15,891–$23,467) in the Pacific Census Division.

During July 1, 2016–February 7, 2020, a total of 32 states reported >18,900 outbreak-associated hepatitis A hospitalizations resulting from the ongoing hepatitis A outbreaks (*2*). On the basis of results of our analysis as a multiplier, we estimate that hospitalization costs associated with these outbreaks have exceeded $306.8 million (SD $11.4 million) as of February 7, 2020.

Because the NIS reports hospital discharges rather than unique patients, we were unable to identify patients with multiple hospitalizations or estimate the per-person costs of hepatitis A inpatient care. We were also not able to separately report the costs associated with liver transplantation.

Even though using highly sensitive inclusion criteria might have introduced an element of cost overestimation in some patients incidentally diagnosed with hepatitis A while admitted for other conditions, our results almost certainly underestimate hospitalization costs associated with the ongoing hepatitis A outbreaks because NIS does not include hospital-based physician fees. Moreover, the national $306.8 million estimate does not account for outpatient visits, emergency department visits that did not result in an admission to the same hospital, lost productivity, out-of-pocket costs to patients or their informal caregivers, or public health costs associated with the hepatitis A outbreaks, further reinforcing the conservative nature of this estimate.

Given the high proportion of hospitalized patients during the ongoing hepatitis A outbreaks, we estimated the average hepatitis A–related hospitalization costs to highlight the preventable economic burden of these outbreaks on healthcare systems and state governments. Hepatitis A is a vaccine-preventable disease. Despite longstanding vaccination recommendations for adults at increased risk for hepatitis A virus infection or adverse consequences of infection, self-reported adult hepatitis A vaccination coverage with >2 doses was only 10.9% for persons >19 years of age in 2017 (*6*). Our findings underscore the importance of improving hepatitis A vaccination coverage among at-risk adults, in accordance with Advisory Committee on Immunization Practices recommendations (*7*).
